# SUGAR: graphical user interface-based data refiner for high-throughput DNA sequencing

**DOI:** 10.1186/1471-2164-15-664

**Published:** 2014-08-08

**Authors:** Yukuto Sato, Kaname Kojima, Naoki Nariai, Yumi Yamaguchi-Kabata, Yosuke Kawai, Mamoru Takahashi, Takahiro Mimori, Masao Nagasaki

**Affiliations:** Department of Integrative Genomics, Tohoku Medial Megabank Organization, Tohoku University, 2–1 Seiryo-machi, Aoba-ku Sendai, Miyagi, 980-8573 Japan

**Keywords:** Automated analysis, Data cleaning, Illumina HiSeq, MiSeq, NGS

## Abstract

**Background:**

Next-generation sequencers (NGSs) have become one of the main tools for current biology. To obtain useful insights from the NGS data, it is essential to control low-quality portions of the data affected by technical errors such as air bubbles in sequencing fluidics.

**Results:**

We develop a software SUGAR (subtile-based GUI-assisted refiner) which can handle ultra-high-throughput data with user-friendly graphical user interface (GUI) and interactive analysis capability. The SUGAR generates high-resolution quality heatmaps of the flowcell, enabling users to find possible signals of technical errors during the sequencing. The sequencing data generated from the error-affected regions of a flowcell can be selectively removed by automated analysis or GUI-assisted operations implemented in the SUGAR. The automated data-cleaning function based on sequence read quality (Phred) scores was applied to a public whole human genome sequencing data and we proved the overall mapping quality was improved.

**Conclusion:**

The detailed data evaluation and cleaning enabled by SUGAR would reduce technical problems in sequence read mapping, improving subsequent variant analysis that require high-quality sequence data and mapping results. Therefore, the software will be especially useful to control the quality of variant calls to the low population cells, *e.g.*, cancers, in a sample with technical errors of sequencing procedures.

## Background

Next-generation sequencers (NGSs) have become one of the main tools for current biology, being used in the analyses of genomic sequences, gene expressions, and promoter activities. To extract valuable insights from NGSs, it is essential to obtain high quality sequencing data. However, the quality is sometimes affected by errors during sequencing procedures. In the Illumina HiSeq and MiSeq machines, experimental small problems like air bubbles and water condensation in the flowcell are known to reduce sequencing qualities [1].

It is usually difficult to avoid accidental and stochastic occurrence of such technical errors in sequencing procedures. Even though on average the obtained sequences were high-quality, it may also contain low-quality sequences generated from physical portions of the flowcell (tiles) affected by the technical errors. Finding and cleaning such error-affected tiles from the whole data would be useful to obtain reliable results and insights from the NGS data analyses.

Here, we develop a graphical user interface (GUI)-based software SUGAR (subtile-based GUI-assisted refiner). This enables rapid evaluation and cleaning of the Illumina HiSeq and MiSeq data, specifically considering technical errors in flowcells and sequencing run. Novelty of this software includes three points. First, SUGAR is capable of analyzing whole data generated by ultra-high-throughput HiSeq machine. The full data of a HiSeq run (>100Gb) cannot be handled by existing quality control software like TileQC, SolexaQA, and HTQC [1–3] because of memory and/or computational errors. Second, SUGAR generates high resolution heatmaps showing spatial distributions of base quality, read density, and mapping quality scores on the flowcell. These heatmaps visualize low-quality spots of the flowcell that may be affected by technical errors due to air bubbles or other factors, notifying users to improve conditions of sequencing experiments. Third, not only to provide a quality assessment report [4], SUGAR also removes the low-quality read data or changes the low-quality nucleotide calls to N bases that were sequenced in the error-affected spots of the flowcell, by both manual operation and automated analysis using GUI guides. Such data cleaning enabled by the SUGAR would improve quality of sequence read mapping and downstream analyses.

## Implementation

### Running environment and input data formats

SUGAR is implemented in Java as an extended version of the quality control software FastQC [5], and runs on any operating system with the Java Runtime Environment. The users can operate and control the SUGAR with user-friendly GUI that offers interactive analysis capability. The “FastQC-style” GUI also reduces the effort required for initial learning process by new users. The SUGAR can handle following types of sequence data as input file: Fastq [6], Sequence Alignment/Map (SAM) [7], and Binary Alignment/Map (BAM) [7]. Reference sequence file is not required when BAM/SAM files are analyzed.

### Heatmap generation

**Figure 1:**
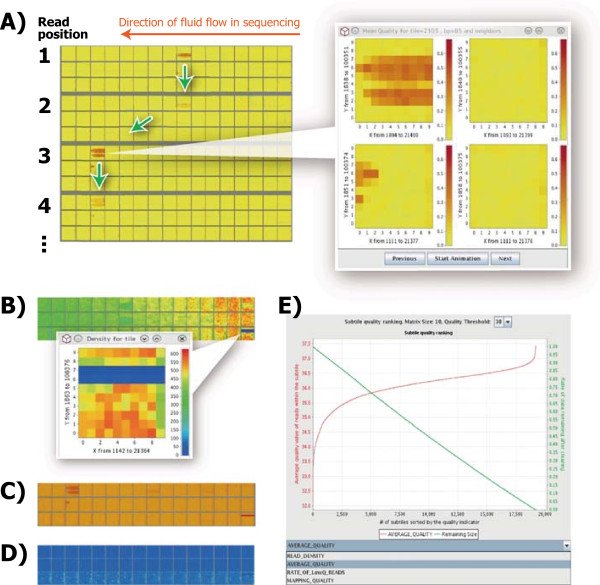
**Heatmap and curve chart for quality assessments. (A)** Subtiles having high proportion of low-quality reads are colored in red. The popup window shows the detailed quality distribution. Green arrows indicate a possible trajectory of movement of the low-quality spots. **(B)** Subtiles having lower density of reads are shown in cold colors. The blue-colored region seems to suffer from a technical error. **(C)** Subtiles are colored by average QV of reads. **(D)** Subtiles are colored by MapQ scores of reads. **(E)** Green curve shows predicted amount of data remaining after automated data deletion from the low-quality subtiles using a given quality threshold indicated by the red curve. Subtiles are ordered along the horizontal axis from low- to high-quality.

From the input file, the SUGAR loads X-Y coordinates, tile number, base quality values (QV) [8], and mapping quality (MapQ) of sequence reads. Then it generates high-resolution heatmaps to show overall distribution of sequencing qualities on the Illumina flowcell (Figure 1). A lane of the flowcell is divided into tiles, which correspond to the scopes of image scanning in the nucleotide sequencing process [9]. For instance, a lane of the MiSeq version 2 and HiSeq2500 Rapid Run flowcells is comprised of 28 and 64 tiles, respectively. Then each tile is further divided into 100 (10×10 resolution) subtiles in a default setting of SUGAR analysis. These subtiles are used as a unit of data quality assessment, and the resultant scores of each subtile are shown as colored dots that constitute the heatmap. Consequently, the heatmap reflects spatial organization of sequencing clusters and their qualities on the flowcell. Resolution of the heatmap (numbers of subtiles/dots) can be changed, although higher resolution requires more memory space. SUGAR also has a downsampling option to conduct quick and rough evaluation of data quality.

### Quality assessments

Overall quality of sequence reads within each subtile is evaluated based on four measures: (1) proportion of low-quality reads in the subtile (Figure 1A), (2) number of reads sequenced in the subtile (read density) (Figure 1B), (3) average QV of the reads in the subtile (Figure 1C), (4) proportion of reads showing low MapQ-scores in the subtile (Figure 1D). Threshold QV to specify data of low-quality (<30 in a default setting) can be changed. These heatmaps enable users to find possible technical errors in sequencing processes. Particularly in result tabs and detailed popup windows of the modules of “proportion of low-quality reads” (Figure 1A) and “average QV of the reads” (Figure 1C), a weighting for heatmap representation between top- and bottom-tiles can be changed by manual operations. This enables virtually three-dimensional evaluation of the distribution of low-quality spots in the flowcell to infer whether the cause of low-quality portion is three-dimensional phenomenon (*e.g.*, air bubbles or debris in sequencing fluids) or two-dimensional phenomenon (*e.g.*, cracks on a flowcell or imaging errors).

### Parameter setting and results evaluation

In the parameter setting of the “proportion of low-quality reads” (Figure 1A), threshold value of 20 in Phred score provides clear visualization result of the heatmaps according to our empirical tests. If an overall quality of the run was remarkably high (which would be checked by Illumina BaseSpace console or the FastQC software), the above threshold can be set to higher one (*e.g.*, 30). If the overall quality was low, the threshold value can be set to lower one (*e.g.*, 10). These parameter changes may show air bubbles or debris on a flowcell more clearly. The heatmaps of “average QV of the reads” (Figure 1C) provide supportive information to quality evaluation by “proportion of low-quality reads” (Figure 1A), in which quality scores of high-quality reads and their variations are not considered and represented in the heatmap.

The read density heatmaps (Figure 1B) show condensation distribution of the reads on a flowcell. Read-dense regions generate greater number of reads with lower quality, while read-sparse regions generate less number of reads with higher quality, in general. By comparing the read-quality heatmaps with the read-density heatmaps, the users can examine whether the low-quality regions are related to read densities and DNA concentration loaded on a flowcell, providing possible feedback to the improvement of DNA experiments. The mapping quality heatmaps (Figure 1D) enable the users to examine whether or not the detected variants came from low-quality regions of a flowcell. This type of analysis has not been provided by other quality-control softwares, however, it would be particularly useful for careful examination of mutation finding from a high coverage data, to improve analyses of, *e.g.*, somatic mutations, cancer cells, or mitochondrial heteroplasmy.

### Predictions of data cleaning results

SUGAR also generates curve charts to predict remaining amount of data after removing low-quality tiles/subtiles (Figure 1E). In these charts, subtiles are ordered and positioned along the horizontal axis on the basis of four types of quality indicator following: (1) read density, (2) average QV, (3) proportion of low-quality reads, and (4) average MapQ, any of which users can choose to generate the graph. Values of selected quality indicators are plotted as red curve. Green curve shows predicted amount of data that remains after discarding the subtiles with given thresh-olds of the quality indicator shown by red curve.

### Removing low-quality tile/subtile and data outputs

SUGAR conducts data cleaning via both manual and automated operations. In the former manual approach using the GUI, the users can select low-quality tiles/subtiles to discard the reads within those regions, or select low-quality nucleotide positions in the tiles/subtiles to change the unreliable nucleotide calls to N bases from the original data. In the latter mode, SUGAR automatically removes reads or changes nucleotides to N-base within low-quality tiles/subtiles. The threshold QV and remaining amount of the data can be specified from the curve charts with GUI guide (Figure 1E).

## Results and Discussion

The SUGAR successfully generated high resolution heatmaps from a human genome sequencing data (Figure 1; 10×10 resolution). We conducted this performance test using a HiSeq2000 data of HapMap individual NA12877 (ERA172924; read length of 100 bp and read coverage of 60× on average). The Fastq sequence reads were mapped to reference genome GRCh37 by the BWA [10]. The obtained 130GB BAM file was analyzed by SUGAR with computing environment with the Intel Xeon CPU E5-2640 processors (2.50 GHz). The CPU time and maximum memory size were less than 5 hours and 250 MB, respectively. An analysis of the corresponding Fastq file is much faster, generally finishes within an hour. Benchmark test was omitted in this study, because the existing tile-based quality control tools were not capable of processing whole HiSeq data using the computing environment described above.

The resultant heatmaps and curve chart-based data cleaning appear to contribute to improving both sequencing procedure and resultant data quality. First, the heatmaps indicate technical errors possibly arose from air bubbles (Figure 1A) and flowcell crack or primary data-processing error (Figure 1B). Such information would be useful to improve NGS machine conditions and sequencing workflows. Second, an average MapQ score of a HapMap individual NA12878 data (SRP021027; 100 bp data by the HiSeq2500; mapped by the Bowtie2 [11]) was increased from 29.5 to 30.1 after an automatic subtile deletions by applying the average QV threshold of 22 (>70% of data remains; Figure 1E). The discarded read data showed an average MapQ of 25.0. Although a conventional read quality-based data filtering provides improved MapQ score (*e.g.*, 35.1 when the lower 30% reads were discarded from the same data set), a combination of such read-based filtering with the tile-based data cleaning of SUGAR provides higher MapQ score (*e.g.*, 35.3 when the tile-based and read-based filtering were applied to the same data set). These results imply that low-quality subtiles may reduce an overall quality of the results of NGS data analyses, particularly in the case of low quality data sets, and thus the subtile-based data cleaning potentially has significant consequences. The detailed data evaluation and cleaning enabled by the SUGAR would reduce technical problems in sequence read mapping, improving subsequent analyses such as somatic mutation identification that require high-quality sequence data and mapping results. Taken together, we believe that the SUGAR, a GUI-based user-friendly tool, would contribute to controlling quality and reliability of upcoming high-throughput omics studies.

## Conclusions

We developed a platform-independent java software SUGAR (subtile-based GUI-assisted refiner) to conduct quality evaluation and the quality-based cleaning of full HiSeq and MiSeq data through visualizing data quality distribution on the flowcell. The analysis can be done with user-friendly, GUI-based operations with relatively quick information processing and low memory space requirements (up to 250 MB for one data set). SUGAR will be especially useful to control the quality of variant calls of a sample with technical errors in sequencing procedures.

## Availability and requirements

**Project name:** SUGAR.

**Project home page:**https://github.com/biomedinfo/sugar.

**Operating system(s):** Platform independent.

**Programming language:** Java.

**Other requirements:** Java version 6 or later.

**License:** GNU GPL version 3 or later.

## Abbreviations

CPU: Central processing unit
GB: Gigabytes
Gb: Giga base pairs
GHz: Giga hertz
GUI: Graphical user interface
MapQ: Mapping quality
MB: Megabytes
QV: Quality values.
